# Sepsis-Induced Cardiomyopathy: Oxidative Implications in the Initiation and Resolution of the Damage

**DOI:** 10.1155/2017/7393525

**Published:** 2017-09-19

**Authors:** Vasiliki Tsolaki, Demosthenes Makris, Konstantinos Mantzarlis, Epameinontas Zakynthinos

**Affiliations:** Department of Critical Care, University Hospital of Larissa, University of Thessaly Medical School, Larissa, Greece

## Abstract

Cardiac dysfunction may complicate the course of severe sepsis and septic shock with significant implications for patient's survival. The basic pathophysiologic mechanisms leading to septic cardiomyopathy have not been fully clarified until now. Disease-specific treatment is lacking, and care is still based on supportive modalities. Septic state causes destruction of redox balance in many cell types, cardiomyocytes included. The production of reactive oxygen and nitrogen species is increased, and natural antioxidant systems fail to counterbalance the overwhelming generation of free radicals. Reactive species interfere with many basic cell functions, mainly through destruction of protein, lipid, and nucleic acid integrity, compromising enzyme function, mitochondrial structure and performance, and intracellular signaling, all leading to cardiac contractile failure. Takotsubo cardiomyopathy may result from oxidative imbalance. This review will address the multiple aspects of cardiomyocyte bioenergetic failure in sepsis and discuss potential therapeutic interventions.

## 1. Introduction

Myocardial depression may develop in patients with severe sepsis and septic shock, complicating the course of their disease. There are reports that it may develop in nearly 60% of septic patients [1]. Parker et al. were the first to describe this entity in 1984 [2]. Sepsis-induced cardiomyopathy is characterized by the presence of left ventricular dilation with normal or low filling pressures and decreased ejection fraction. Characteristically, the syndrome has a reversible character, beginning to normalize within 7–10 days of onset [3]. Importantly, sepsis-induced cardiac dysfunction has a negative impact on patient's survival [4].

The exact pathophysiologic mechanisms, ultimately leading to cardiac dysfunction, are not well clarified. Endotoxins and inflammatory cytokines seem to play a key role in the genesis of myocardial depression. Moreover, hypoxia and acidosis, hypotension and hypovolemia, metabolic disturbances, coagulation abnormalities, and increased production of reactive oxygen and nitrogen species (ROS and RNS) have been proposed to participate in myocardial depression during sepsis [5]. On the other hand, hypotension/hypoperfusion does not appear to be the key mechanism in the genesis of myocardial dysfunction. Measurements of coronary artery-coronary sinus oxygen content difference revealed a reduced value, pointing out the oxygen utilization problem rather than oxygen delivery [6]. In the late phases of sepsis, tissue oxygen tension is increased emphasizing that the major problem is oxygen utilization, a condition known as cytopathic hypoxia [7]. The majority of body oxygen is taken up by the mitochondria and is used for energy production (in the form of adenosine triphosphate (ATP)). Thus, it appears that these organelles may play a pivotal role in the pathogenesis of sepsis-induced organ dysfunction. Oxidative stress has well been implicated in sepsis in humans, having been found to correlate with the severity of the disease and mortality [8, 9]. Reactive oxygen and nitrogen species are produced in excess and have been implicated in the genesis of sepsis-induced myocardial dysfunction [10–16]. Imbalance in oxidative status leading to overproduction of reactive oxygen species (ROS) on the one hand and nitric oxide (NO) and its toxic derivative, peroxynitrite, on the other are major contributors to myocardial injury [15, 17].

In the present review, we will focus on the role of reactive oxygen and nitrogen species in the generation of myocardial dysfunction in sepsis. Moreover, we will review current treatment options targeting oxidative stress imbalance, responsible for cardiomyopathy in sepsis.

### 1.1. Search Strategy

Data for this review were collected by searching PubMed and from references of the related articles. We attempted a comprehensive research in PubMed, through April 2017, using the terms “septic cardiomyopathy and reactive oxygen species,” “septic cardiomyopathy and mitochondrial dysfunction,” “reactive oxygen species and heart and sepsis,” “redox and heart and sepsis,” “mitochondria and heart and sepsis,” and “energy metabolism and heart and sepsis.” The search was limited to publications in English. In addition, we searched the online registry of randomized controlled trials of the US National Institutes of Health (http://www.clinicaltrials.gov) and the Current Controlled Trials website (http://www.isrctn.com) for ongoing investigations regarding this subject using the aforementioned terms. Seven hundred and ninety studies were initially found: 667 of them were excluded after abstract review because they were irrelevant. We focused on the rest 127 clinical studies which evaluated the relationship between oxidative stress and the myocardial dysfunction in sepsis.

## 2. Reactive Species

There is evidence that during sepsis, there is increased production of free radicals from cardiomyocyte mitochondria which depresses myocardial function. A free radical is a molecule characterized by the presence of one or more free electrons in the outer orbit. The presence of these electrons gives the molecule great instability, making it highly reactive and toxic. The reactivity of different free radicals varies, but some can cause severe damage to biological molecules, especially to DNA, lipids, and proteins [18]. Oxygen containing free radical molecules and their precursors formed in biological systems are collectively termed reactive oxygen species (ROS), including superoxide (O_2_^−^), hydrogen peroxide (H_2_O_2_), and hydroxyl radical (OH). On the other hand, NO produced from nitric oxide synthases (NOS) may react with free radical of oxygen forming peroxynitrite (ONOO^−^), a molecule supposed to be the NO toxicity mediator, exhibiting multiple inhibitory actions in the mitochondrial respiratory chain [19, 20]. In a redox balance, reactive species play an important role in the life cycle of cells, the induction of cell signaling pathways, the activation of intra- and intercellular secondary messengers, and immune cell defense mechanisms. RNS are also involved in the regulation of blood pressure and vascular tone, activation of NF-kB, release of inflammatory cytokines, and expression of adhesion molecules [18, 21–23]. Oxidative stress occurs when the production of ROS and RNS lays beyond antioxidant protection mechanisms, leading to mitochondrial failure.

Mitochondria, which are placed in the cytoplasm of the cardiomyocyte, are the main sources of energy supply through oxidative phosphorylation. Sepsis diminishes the total capacity of the respiratory chain leading to energy imbalance. During oxidative phosphorylation, a small amount of O_2_^−^ (superoxide anion) is produced, which is scavenged to generate H_2_O_2_ by Mn-superoxide dismutase (MnSOD), one of the major antioxidant systems in cells. On the other hand, mitochondria are one of the major organelles that initiate and sustain energy imbalance in the cardiomyocyte during sepsis, leading to myocardial dysfunction [24].

### 2.1. ROS Production in Septic Hearts

**Figure 1:**
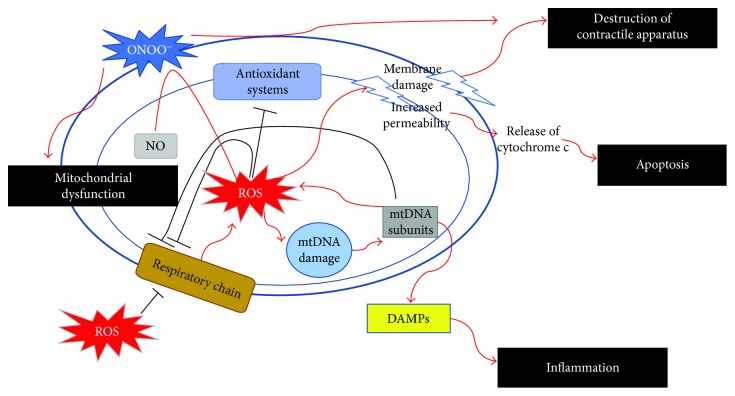
Oxidative damage in septic cardiomyocytes. In the presence of ROS, electron flow through the respiratory chain is impaired leading to further production of ROS. Mitochondrial ROS production leads to oxidative damage to proteins, lipids, and DNA subsequently leading to further mitochondrial dysfunction, apoptosis, destruction of the contractile apparatus, and promoting inflammation. NO reacts with ROS to generate ONOO^−^, the cytotoxic product of NO. ROS: reactive oxygen species; mtDNA: mitochondrial DNA; DAMPs: danger-associated molecular patterns; NO: nitric oxide; ONOO^−^: peroxynitrite.

Endotoxins, produced during sepsis, are capable of inducing ROS production by the mitochondria [25, 26]. In cardiomyocytes, endotoxin has been shown to induce the production of superoxide, hydrogen peroxide, and hydroxyl radical through xanthine oxidase, NADH/NADPH oxidases, and mitochondria [27–29]. ROS generation by the mitochondria further stimulates ROS production in endothelial cells, triggering a vicious cycle of free radical production resulting in a wide variety of reversible and irreversible toxic modifications on biomolecules [30–32]. ROS production leads to ultrastructural and functional changes in mitochondria, some of them being reversible during the recovery phase of sepsis, others causing irreversible mitochondrial failure leading to multiple organ dysfunctions (mechanisms summarized in Figure 1) [33–35]. NADPH oxidases consist of a membrane-bound catalytic subunit (NOX) and a number of cytosolic regulatory subunits, which have been found to increase their activity in response to sepsis (lipopolysaccharides, LPS) [28, 36, 37]. Enhanced NO and superoxide production and thus peroxynitrite occur in dysfunctional hearts from rats, while increased levels of the lipid peroxidation product, malondialdeyde (MDA), have also been found indicating the role of underlying oxidative stress in septic hearts [35, 38]. Moreover, using an animal model, it was shown that ROS production correlated with the increase of a NADPH subunit (NOX_1_) mRNA, leading to increased cardiomyocyte apoptosis, while animals deficient of this subunit did not present increased rates of apoptosis. Furthermore, the same study showed that after LPS infusion, there was a significant reduction in heart performance (indicated by the fraction shortening (%FS)), as well as an increase in left ventricular end systolic diameter, indicating myocardial contractile dysfunction [39]. Noteworthy, there is evidence suggesting that the severity of mitochondrial dysfunction correlates with the severity of sepsis [37].

#### 2.1.1. ROS Production Affects Proteins, Lipids, and DNA

*(1) Enzyme Dysfunction*. ROS and RNS lead to lipid peroxidation, protein oxidation, and nitration and DNA fragmentation. Therefore, oxidative stress imbalance may compromise the integrity of cell membranes and may affect enzyme function and gene expression. ROS and RNP overproduction by the mitochondria has been found to inhibit oxidative phosphorylation, thus resulting in decreased production of ATP [40]. The state where mitochondria cannot utilize the delivered oxygen is known as “cytopathic hypoxia.” This condition is verified by the decreased oxygen content difference between the coronary arteries and coronary sinus, previously found (indicating reduced cell utilization of oxygen in the abundant presence of oxygen molecules). “Cytopathic hypoxia” is the most important step in the development of multiorgan failure in sepsis [6, 41, 42]. Endotoxin administration in animals results in reduction in mitochondrial state 3 respiration rate and reduces ATP production, while oxygen consumption decreases. All these result in reduced cardiac pressure-generating capacity [38]. Animal models with sepsis presented inhibited electron flow through complexes I, II, and III of the electron transport chain and inhibition of oxidative phosphorylation and ATP generation [35]. Moreover, mitochondrial dysfunction alters myocyte contractility and its electrical properties and leads eventually to cell death [43]. Smeding et al., reviewing literature on cardiac structural alterations during sepsis, concluded that the impairment in cardiac mitochondrial function correlated with decreased cardiac contractility [44]. Matkovich et al. showed that there is wide downregulation (up to 50%) of cardiac mitochondrial genes during sepsis, with the majority of genes coding for members of the electron transport chain and almost every step of the Krebs tricarboxylic acid. Interestingly, they also found decreased expression levels of genes encoding major proteins of the cardiac sarcomere and the excitation-contraction coupling process in septic cardiomyopathy [45].

Oxidative and nitrative stress can lead to activation of the nuclear enzyme poly (adenosine 5′-diphosphate [ADP]-ribose) polymerase (PARP) with subsequent loss of left ventricular systolic work index [46].

*(2) Lipid and Protein Oxidation*. ROS overproduction induces lipid oxidation, further compromising the integrity of membranes. In a time-course study in a rat model of pneumonia-related sepsis, Zang et al. found that sepsis produces progressive oxidative mitochondrial damage in the heart, as confirmed by mitochondrial outer membrane damage and release of cytochrome c [47]. They also found greater lipid and protein oxidation, following downregulation of the activities of antioxidant enzyme SOD and GPx in mitochondria [47]. Cytochrome c release from mitochondria is initiated by ROS-mediated peroxidation of cardiolipin, a phospholipid component of the mitochondrial inner membrane [48]. Cardiolipin oxidation leads to mitochondrial transition pore opening and dissociation of cytochrome c. Cytochrome c is released to the cytosol, activating caspase 9 and subsequently caspase 3 and 7, being responsible for the biochemical and morphological change characteristic of apoptosis [49–51].

Oxidative damage to lipids and proteins is responsible for structural myocardial changes and responsible for the clinical presentation of septic cardiomyopathy. These changes seem to precede phenotypic changes that characterize septic cardiomyopathy. There is evidence of sarcolemma damage leading to increased plasma membrane permeability as an early event in cecal ligation and puncture- (CLP-) induced severe sepsis in mice [14]. Increased sarcolemma permeability indicates functional impairment of the dystrophin glycoprotein complex (DGC) in severe sepsis. Of the DGC proteins, dystrophin forms a strong mechanical link between the sarcolemma and costameric cytoskeleton in the cardiac muscle cells, providing structural stability to the cell membrane and the sarcolemma against stresses generated during muscle contraction [52]. Furthermore, sepsis mitochondrial damage is probably the triggering factor leading to myocardic cell vacuolation indicating apoptosis in the myocardium [53]. Other studies have confirmed loss of mitochondrial structure integrity, as a response to septic stimuli, such as derangements of mitochondrial cristae and mitochondrial matrix edema [46, 54].

*(3) ROS Production Initiates Inflammatory Responses*. Mitochondrial damage resulting from imbalanced production of ROS (mtROS) impairs mitochondrial structure on the one hand and mitochondrial biogenesis via oxidative modifications on macromolecules, such as mitochondrial DNA (mtDNA), on the other. Mitochondrial structures (mtROS, mtDNA, cytochrome c, ATP, and cardiolipin) are increasingly recognized as regulators/promoters of inflammation, as they function as danger-associated molecular patterns (DAMPs) (mechanisms summarized in Figure 1) [55–61]. In animal models, it was seen that after the induction of sepsis, almost half of mtDNA loses its integrity, being dependent on mtROS signaling. MtROS mediate reduction of mitochondria-located mtDNA repair enzymes, also inducing mitochondrial functional deficiency and structural impairment in the heart. In the same study, mtROS-mediated mtDNA damage increased the expression of MYD88 and RAGE, both being implicated in promoting cytokine production through a cytosolic DNA-TLR9-dependent signaling pathway, inducing downstream inflammatory responses [62].

Mitochondria's implication in the regulation of inflammation is assumed by the activation of nuclear factor-kappa B (NF-kB), a crucial mediator of apoptosis. Activation of NF-kB is associated with its translocation from the cytosol to the nucleus. Septic challenge decreases cytosolic with a simultaneous increase of nucleic NF-kB, indicating activation of this molecule [47, 53]. The mitochondrial matrix proteins MAVS and DOC-4 were identified as signaling factors that regulate NF-kB activation, and changes in mitochondrial Ca^2+^ have been suggested to modulate cytokine production in cardiomyocytes [63–65]. Inflammatory reactions in the septic heart increase progressively but not before the changes in cardiac mitochondria. This finding suggests that sepsis produces a cascade of myocardial mitochondrial damage, namely, mitochondrial release of cytochrome c, damage to the mitochondrial outer membrane, increase in lipid and protein oxidation, and decrease in mitochondrial ROS defenses, followed by progressive myocardial inflammation and late cardiac dysfunction [47].

*(4) Intracellular Signaling*. Many extracellular stimuli recognized by cells involve a complex intracellular signaling network; the most important of which being mitogen-activated protein kinases (MAPK). MAPK are sensitive to reactive oxygen species, being activated by NADPH oxidase [66]. The most extensively studied members of the MAPK are extracellular signal-regulated kinase 1/2 (ERK 1/2), p38 MAPK, and c-Jun N-terminal kinase (JNK). Activation of these members has been found to be implicated in the genesis of circulatory shock, while, via activation of other inflammatory enzymes (such as COX-2), overproduction of prostanoids may be involved in the myocardial dysfunction associated with sepsis [67–69].

### 2.2. Role of NO and Peroxynitrite

The role of high NO concentration in the septic heart is still, as yet, controversial. NO is an important bioactive substance which plays an important role in the regulation of normal body function and disease occurrence. It is thought of as a signaling molecule with a multitude of biological actions and targets. It has a half-life of a few seconds and is produced in many cell types within the heart [70]. NO synthesis is activated by one of the three isoforms of NOS that catalyze NADPH-dependent oxidation of l-arginine to NO and l-citrulline: NOS_1_ (neuronal or nNOS), NOS_2_ (inducible or iNOS), and NOS_3_ (endothelial or eNOS) [71]. All three isoforms are found in cardiomyocytes. NO plays multiple roles in cardiac physiology in health and disease [70]. It results in vasodilation (including coronary arteries), suppresses mitochondrial respiration (regulatory control), and regulates the release of proinflammatory cytokines. It regulates adhesion and aggregation of platelets and smooth muscle cell proliferation, thus functioning as a cardioprotective substance [72–74]. Apart from coronary vasodilation, NO may increase ventricular compliance, resulting in increased cardiac preload and myocardial blood supply [75]. Furthermore, NO may serve to restore myocardial function by promoting de novo synthesis of mitochondrial proteins. Additionally, by reducing oxygen consumption, NO preserves calcium sensitivity and contractile function, contributing to hibernation in response to myocardial ischemia [76, 77]. However, excessive formation of NO plays a central role in septic shock and has been found to contribute to contractile dysfunction [4, 78]. Increased oxidative stress, impairment in oxidative phosphorylation function, and a decrease in ATP production were restored by genetic deletion of iNOS (iNOS −/− mice). Moreover, inhibition of iNOS by melatonin prevented the impairment of mitochondrial homeostasis after sepsis and, finally, improved survival [79, 80].

Studies of animals subjected to endotoxemia have demonstrated that NO production, production of O_2_^−^ and H_2_O_2_, global protein nitration, nitrotyrosine content, protein carbonylation, and lipid peroxidation are increased in cardiac mitochondria [81–83]. Nitric oxide (NO) and superoxide (O_2_^−^) rapidly react to form the toxic product peroxynitrite anion (ONOO^−^) [84]. ONOO^−^ is a crucial pathophysiological event which occurs during sepsis, since it represents a critical cytotoxic factor in oxidative stress-mediated tissue damage, supposed to be the NO toxicity mediator, inhibiting in multiple ways the mitochondrial respiratory chain [19, 20].

Peroxynitrite is able to enter the cell membrane and consequently oxidize multiple target molecules, either directly or through the generation of reactive radicals, resulting in structural modification and dysfunction of lipids, proteins, and nucleic acids. They can disrupt DNA integrity, impair the activity of ion channels, break down the mitochondrial respiratory chain, and induce cell death [85]. Several animal models have demonstrated the implication of peroxynitrite in sepsis and in septic cardiomyopathy as well. It has been shown that LPS-treated mice are under oxidative stress and reactive oxidative species, such as superoxide, and peroxynitrite is mainly involved in the oxidative stress formation [86]. Endotoxemic shock is accompanied by a marked increase in mtNOS activity in the heart, leading to increased production of NO, O_2_^−^, H_2_O_2_, and ONOO^−^, causing mitochondrial dysfunction and contractile failure [87]. Many of these oxidative radicals such as superoxide, nitric oxide, and peroxynitrite have been demonstrated in septic hearts in animal models, whereas the latter may be responsible for cardiovascular alterations met in septic shock, such as vascular hyporeactivity, myocardial impairment, and energetic failure [88]. Moreover, increased expression of peroxynitrite participates in the fall of blood pressure, endothelial injury, multiple organ dysfunction, and subsequent death, as was depicted in rats treated with LPS [82, 83, 89]. Endogenous formation of peroxynitrite induces cytotoxic effects in myocardic cells, which, in turn, decreases the ability of the heart to convert ATP into mechanical work, leading to myocardial contractile dysfunction [90] (mechanisms summarized in Figure 1).

Recently, it was shown that animals with preexisting cardiac disease (atherosclerosis) presented impaired ventricular dilation (the relaxation time constant *τ* decreased while dp/dt_max_ increased) and preserved systolic function (unchanged ejection fraction), after the induction of faecal peritonitis. Cardiac nitrotyrosine formation, a well-established marker for both augmented oxidative and nitrosative stress, increased [91].

In humans, there is evidence for significant presence of peroxynitrite in myocardial specimens from septic patients who have died, and it has been shown that septic hearts demonstrate peroxynitrite-induced protein nitration and activation of the proteolytic ubiquitin-proteasome pathway [92–94]. The most abundant proteins for nitration modification within cardiac myocyte are actin and myosin. The observation of scattered foci of actin and myosin filament disruption in septic hearts supports the idea that tyrosine nitration could potentially decrease myocardial contractility by directly modifying the contractile apparatus [93]. In another series of biopsies obtained from septic patients, it was shown that peroxynitrite is overproduced in the heart of septic but not control patients and the inducible isoform of NOS (NOS-2) is overexpressed in the left as well as the right ventricle and both atria. This study also demonstrated that peroxynitrite-induced tyrosine nitration, which has been shown to alter contractile protein function, leads to contraction and relaxation alterations in septic hearts [92].

### 2.3. Takotsubo Cardiomyopathy

Takotsubo cardiomyopathy, also known as stress-induced cardiomyopathy, is an acute syndrome characterized by reversible wall-motion abnormalities, triggered by an emotional or physical stressor, occurring in acute medical illness, such as sepsis, trauma, intracerebral haemorrhage or even postpartum [95–98]. The exact pathophysiological mechanisms leading to this entity are not well clarified, with catecholamines being the most appealing explanation leading to myocardial stunning [99]. Oxidative stress is a rising, not thoroughly evaluated, pathogenetic mechanism, implicated in the pathophysiology of the syndrome. Upregulation of HO-1 was observed in an animal model of stress-induced takotsubo cardiomyopathy. Cardiac-specific induction of OH-1 is cytoprotective against oxidative stress and has been found to restore ventricular function, protecting tissue from ischemia/reperfusion injury and postmyocardial infarct remodelling [100–102]. In takotsubo cardiomyopathy, isolated hearts show impaired contractile-metabolic coupling, while there is an altered mitochondrial oxidative metabolic state, increased mitochondrial fragility, and oxidative stress. Interestingly, there was a noted decrease in the activities of respiratory chain complexes I and II (as high as 65 and 82%, respectively, in state 3) [103].

### 2.4. Antioxidant Reserve

The term antioxidant is vaguely defined in the literature and, according to its use, can refer to an array of compounds with varying mechanisms of action [104]. One proposed definition emphasizes that “an antioxidant is any substance that, when present at concentrations lower than those of an oxidizable substrate, significantly delays or prevents oxidation of that substrate” [105]. Mitochondria are protected from damage caused by ROS, through several antioxidant systems. When ROS production exceeds antioxidant protection mechanisms, oxidative stress damages nitric acids, proteins, and lipids in mitochondria, ultimately leading to impairment of ATP production through loss of enzyme function in the energy transport chain (ETC) [106]. Antioxidants can be damaged through protein oxidation and peroxidation of cardiolipin (leading to the dissociation of cytochrome c and further generation of ROS) [107]. Antioxidant systems are classified as enzymatic and nonenzymatic as well as endogenous and exogenous. Enzymatic molecules include those that scavenge ROS (superoxide dismutase SOD, glutathione peroxidase (GPx), catalase, and thioredoxin). Among nonenzymatic molecules usually ingested in the diet are vitamins (A, C, and E), amino acids, and metals (copper and selenium) [108]. These mechanisms act synergistically to balance redox overproduction [104].

Intramitochondrial production of NO causes glutathione depletion [19]. Sepsis has been found to increase the activity of enzymes related to the metabolism of glutathione. Tissues are able to increase glutathione levels through de novo synthesis in response to infection, whereas there are other factors that decrease its synthesis during sepsis such as anti-inflammatory cytokines, malnutrition, hyperglycemia, and the administration of erythropoietin, glucocorticoids, and catecholamines [109]. Low glutathione levels are associated with higher mortality in sepsis experimental models, as glutathione is the main mechanism protecting cells from oxidative damage [105].

Cardiac mitochondrial SOD and GPx decrease after sepsis, with SOD activities being reduced 4–8 h after sepsis challenge and GPx activity falling to 70% after 12–24 h [47]. Moreover, glutathione peroxidase, degrading hydrogen peroxide, H_2_O_2_ is found reduced 16 hours after sepsis, with the reduction in the levels coinciding with reduced cardiac contractility [11]. LPS-induced myocardial depression (measured as peak tension generated by myocardial contraction) coincides with decreased activity of GPx, the most abundant antioxidant enzyme in myocardium, and decreased levels of GSH, the most important thiol in combating oxidative stress [11]. Moreover, strain echocardiography identified septic cardiomyopathy which correlates with reduced expression of key mitochondrial ROS scavengers [110]. In animals, it has been found that both superoxide dismutase and glutathione peroxidase activities, in cardiac mitochondria, decrease (as much as 40% and 70% compared to animals without sepsis) early after sepsis induction and remain at lower levels throughout the first 24 hours after LPS challenge. These findings suggest that sepsis depletes mitochondria of their defense mechanisms against ROS [53].

### 2.5. Treatment Implications and Future Directions

Since oxidative damage to mitochondria is central to the pathology of sepsis, antioxidants could be potential therapies in resuscitating mitochondrial function further implementing organ resuscitation. Antioxidants have been used to improve cardiac function in other medical conditions as well [111–113].

#### 2.5.1. Conventional Treatments

Preparation of septic animal models with antioxidants prevents the increase in cardiac mitochondrial generation of reactive oxygen species and, most importantly, prevents reductions in systolic pressure-generating capacity of the septic hearts [38]. Treatment with antioxidant vitamins has been found to improve myocardial contraction and relaxation defects in septic animals as a consequence of alleviated inflammatory response and apoptosis [114]. Naringin, an antioxidant, anti-inflammatory, and antiapoptotic flavanone glycoside found in grapefruits and oranges, when given orally in septic mouse models, regulated the expression and release of superoxide dismutase (SOD) and malondialdehyde (MDA) to inhibit the subsequent myocardial oxidative stress, suppressed myocardial cell apoptosis, and ameliorated heart morphological changes, all these leading to improved mouse survival [115].

Another experimental sepsis model showed that treatment with antioxidant vitamins alleviated both the systemic and myocardial inflammatory cytokine response and that it inhibited NF-kB nuclear translocation, decreasing caspase-3 and caspase-8 myocardial activity, thus decreasing myocardial apoptosis [114]. Other studies have found, in vivo, that antioxidant treatment significantly attenuated the loss of sarcolemma dystrophin expression and the increased plasma membrane permeability [14]. Cardiomyocytes lacking dystrophin are abnormally vulnerable to mechanical stress-induced injury, with loss of sarcolemma integrity and increased fragility and permeability [116, 117].

It has been documented that neutralization of peroxynitrite can reduce its accumulation and improve myocardial contractile dysfunction and inflammation in septic animal models [118, 119]. Peroxynitrite neutralizers can prevent left ventricular systolic function alterations of endotoxin-treated hearts, left ventricular developed pressure, and its maximal first derivatives (i.e., dp/dt). Moreover, they can prevent I-kappa-B degradation and reduce plasma TNF-alpha levels in endotoxin-treated rats, leading to reduced leucocyte infiltration and endothelium-leucocyte activation [119].

#### 2.5.2. Mitochondria-Targeted Antioxidants

Mitochondria-targeted delivery of antioxidants provides mitochondrion-specific antioxidant defense, protects mitochondria from oxidative damage, prevents mitochondrial membrane damage, improves mitochondrial respiratory function in the heart with sepsis, and improves cardiac function in septic animals.

Mitochondria-targeted vitamin E prevented NF-kB activation, suppressed myocardial injury denoted by serum troponin-I (cTnI) levels, and prevented myocardial apoptosis, ameliorating sepsis-induced disorganization and DNA fragmentation. Taken together, these data suggest that targeted suppression of mtROS suppresses cardiac inflammation and improves cardiac performance in sepsis [62]. Mitochondria-targeted vitamin E increased antioxidant capacity in a rat pneumonia-related sepsis model, reduced the leakage of cytochrome c from mitochondria to cytosol, and suppressed sepsis-induced myocardial inflammation, all of them preventing sepsis-induced left ventricular decompensation. Rats receiving mito-Vit E preserved their % EF (ejection fraction) and % FS in contrast to controls [120]. Furthermore, this study provides clear evidence that mitochondria-targeted antioxidant therapy could be more effective in ameliorating oxidative damage and improving organ function in sepsis, than conventional antioxidant therapies, and this is because antioxidants are distributing throughout the body and not accumulating in the mitochondria, where they are mostly needed. Conventional antioxidants may have failed to present significant efficacy due to their low penetrance to the mitochondria interior, where ROS are mainly produced. Mitochondria-targeting antioxidants have been effective in counterbalancing ROS production in other disease states, such as kidney ischemia/reperfusion injury [121].

#### 2.5.3. Other Potential Treatments

Cytochrome oxidase (CcOX), the terminal oxidase of the respiratory chain, uses electrons donated by cytochrome c to reduce oxygen to H_2_O [122]. CcOX inhibition is competitive and reversible early after the induction of sepsis in experimental models, becoming irreversible and noncompetitive during the late phase of sepsis, which is associated with deterioration in myocardial function and survival [123]. It has been shown that exogenous administration of cytochrome c could gain access to cardiomyocyte mitochondria and replete mitochondria with supranormal levels of substrate, thus overcoming competitive inhibition of CcOX. Exogenous administration of cytochrome c improved myocardial contractility and relaxation, as depicted by an increase in left ventricular systolic pressure and a 45% increase in dP/dtmax and dP/dtmin. Importantly, these improvements occurred without significant increases in heart rate, LV end-diastolic pressure, or tau (the LV isovolumic relaxation constant) [124].

Sepsis triggers intracellular signaling cascades, regulated, mainly, by intracellular kinases, phosphorylating downstream targets. Among these, small GTPases of the ras homologous (Rho) family and one of their effectors, RhoA-associated coiled-coli-containing protein kinases (ROCK) are known to act in regulating actin cytoskeleton organization and cell migration. RhoA/ROCK activation plays an essential role in vascular physiology and pathophysiology [125, 126]. Recently, a study trying to evaluate whether activation of RhoA/ROCK pathway could be involved in mitochondrial dysfunction induced by endotoxemia demonstrated that RhoA/ROCK inhibition normalized mitochondrial respiration in LPS heart and reduced proinflammatory and oxidative stress responses, cytoskeleton disorganization, and mitochondrial ultrastructural damage. Additionally, the study revealed that sepsis caused LV contractile dysfunction, while administration of the ROCK inhibitor improved parameters of LV contractile function (LV tension and maximal positive and negative first derivatives of developed tension (dF/dtmax, dF/dtmin)) [127]. Targeting the ROCK pathway in sepsis could have further therapeutic implications in reducing oxidative stress and inflammation via a NO-dependent mechanism [128].

The peroxisome proliferator-activated receptor- (PPAR-) *γ* coactivator-1*α* (PGC-1*α*) and coactivator-1*β* (PGC-1*β*) modulate members of the PPARs, which further regulate mitochondrial energy metabolism and the production of mitochondrial ROS in the heart. Both pathogen-associated molecular patterns (PAMPs) and danger-associated molecular patterns (DAMPs) downregulate PGC-1*α* and PGC-1*β* and cause impaired cardiac energy metabolism [129, 130]. A newly synthetic antimicrobial peptide 19-2.5 (Pep2.5), acting against PAMPs, has been shown to counterbalance mitochondria dysfunction in cardiomyocytes during sepsis. Martin and coworkers showed that Pep2.5 enhances mitochondrial respiration, increases ATP levels, and downregulates the production of mtROS in cardiomyocytes during sepsis, by attenuating the suppression of PPARs and PGC-1*α*/*β* [131].

## 3. Conclusion

Mitochondrial injury and dysfunction are two of the major determinants of a clinical spectrum of phenomena seen in septic patients, called septic cardiomyopathy. Oxidative and nitrosative stress, generated in mitochondria, impairs cardiac contractility during sepsis. Oxidative stress leads to energetic (and thus functional) and structural failure of the cardiomyocyte. On the other hand, during sepsis, there are various mechanisms through which the organism tries to protect itself against energy dysfunction, including a reduction in the basal functions (and therefore the energy requirements) of cells and metabolic pathways, an increase in the consumption of energy reserves, and the activation of damage repair mechanisms. This phenomenon is known as cell hibernation and is an effort to avoid cytopathic hypoxia. There is evidence that cells can and do change their energy metabolism [132, 133].

It appears that inhibition of oxidative stress diminishes myocardial damage. However, once mitochondrial damage has occurred, recovery depends on the efficiency of biogenesis (removal and replacement) of the damaged mitochondria. Current research focuses on mitochondrial dysfunction, and mitochondria-targeted therapies are expected to gain wide acceptance. Mitochondria-targeted antioxidants represent an attractive therapeutic approach for diseases complicated by mitochondrial oxidative damage. In the future, managing energetic failure may be a more efficacious treatment modality, rather than treatments focusing on multiple organ failures.
